# The Single-Cell Sequencing: A Dazzling Light Shining on the Dark Corner of Cancer

**DOI:** 10.3389/fonc.2021.759894

**Published:** 2021-10-21

**Authors:** Jing Li, Nan Yu, Xin Li, Mengna Cui, Qie Guo

**Affiliations:** ^1^ Department of Clinical Pharmacy, The Affiliated Hospital of Qingdao University, Qingdao, China; ^2^ Department of Pharmacy, Qingdao Eighth People’s Hospital, Qingdao, China

**Keywords:** cancer, single-cell sequencing, intratumor heterogeneity, tumor microenvironment, single tumor cell

## Abstract

Tumorigenesis refers to the process of clonal dysplasia that occurs due to the collapse of normal growth regulation in cells caused by the action of various carcinogenic factors. These “successful” tumor cells pass on the genetic templates to their generations in evolutionary terms, but they also constantly adapt to ever-changing host environments. A unique peculiarity known as intratumor heterogeneity (ITH) is extensively involved in tumor development, metastasis, chemoresistance, and immune escape. An understanding of ITH is urgently required to identify the diversity and complexity of the tumor microenvironment (TME), but achieving this understanding has been a challenge. Single-cell sequencing (SCS) is a powerful tool that can gauge the distribution of genomic sequences in a single cell and the genetic variability among tumor cells, which can improve the understanding of ITH. SCS provides fundamental ideas about existing diversity in specific TMEs, thus improving cancer diagnosis and prognosis prediction, as well as improving the monitoring of therapeutic response. Herein, we will discuss advances in SCS and review SCS application in tumors based on current evidence.

## Introduction

It has been stated that inactivation of oncogenes boosts cancer remission, implying that oncogenes are the Achilles’ heel of cancers independent of the cellular heterogeneity remaining within the tumor. The existing model of cancer has kept oncogenes firmly in focus as therapeutic targets and is in agreement with the fact that all cancerous cells carry the same oncogenic genetic lesions (1). However, it is now recognized that tumor cells accumulate genetic alterations as a way of progressively optimizing themselves, resulting in highly diverse phenotypes of cancer cells. This diversity supports the complicated nature of the tumor microenvironment (TME), in which genetically distinct subclonal populations of cells coexist, resulting in intratumor heterogeneity (ITH) (2). Unfortunately, the understanding of ITH in tumor subsets is still poor. It is of utmost priority to reveal the underlying mechanisms promoting tumor development, metastasis, chemoresistance, and immune escape from the perspective of single cells (3, 4). Fortunately, genome, transcriptome, and epigenome profiling of single cells is well underway with the efforts of the Human Genome Project (HGP) and International HapMap Project, the obvious advances in high-throughput biochip technology have also aided these efforts (5). An understanding of ITH will not be delayed with the advent of single-cell sequencing (SCS) technology, which is expected to enable mapping of the “genetic story” of tumor development, metastasis, chemoresistance, and immune escape by providing centralized dynamic genomic DNA and transcriptome RNA data and epigenetic information scattered across single tumor cells (6). Here, we discuss the common themes emerging from initial studies of SCS and then highlight challenges in cancer biology for which single-cell genomics analysis may provide a compelling approach.

## Overview of Single-Cell Sequencing

Benefiting from the advances in next-generation sequencing (NGS) and third-generation sequencing (TGS) technology, genome-wide scanning strategies have gradually evolved from analyses of mixed cell populations to single-cell targeted assessments (7). Accordingly, a novel method of bulk genomic analysis, advertised as SCS or single-cell RNA sequencing (scRNA-seq), by which the gene copy number in a single nucleus could be precisely measured was introduced by the Cold Spring Harbor laboratory and MD Anderson Cancer Center in 2011 (8).

SCS strategies can be divided into three categories: whole-genome sequencing (WGS), whole-transcriptome sequencing (WTS), and epigenetic sequencing (9). In particular, WGS provides a uniform amplification process for assessing genomic sequences in target cells using an exon-trapping method (10). WTS is aimed at all transcripts and is especially suitable for GeneScan assessment of stem cells and early embryonic cells with high heterogeneity (11). Alternatively, epigenetic sequencing can be used to elucidate the unfolding roles of DNA methylation, histone modification and structural and regulatory chromatin loops in transcriptional heterogeneity (12). Optimized SCS-based strategies such as transposase-accessible chromatin with sequencing (ATAC-seq), single-nucleus RNA sequencing (snRNA-seq), single-cell chromatin immunoprecipitation sequencing (scChIP-seq), topographic single-cell sequencing (TSCS), and clustered regularly interspaced short palindromic repeats (CRISPR) droplet sequencing (CROP-seq) have been rapidly developed (13).

## Brief Glance of SCS Launching

Single-cell separation is the first step of SCS and can be accomplished by immunomagnetic separation (IMS), Raman optical tweezers (ROT), fluorescence-activated cell sorting (FACS), microfluidic, and laser-capture microdissection (LCM) sorting (14, 15). Genome-wide nucleotide amplification, including whole-genome amplification (WGA) and whole-transcriptome amplification (WTA), is a straightforward strategy for SCS. Polymerase chain reaction (PCR) derivatives, such as degenerate oligonucleotide-primed PCR (DOP-PCR), multiple displacement amplification (MDA), and multiple annealing- and looping-based amplification cycles (MLBACs), can be used to accomplish WGA (16). Successful WTA depends heavily on *in vitro* transcription (IVT) and RNA amplification mediated by Phi29 DNA polymerase because polyadenylated mRNA is reverse transcribed and then linked to a splice sequence with oligo-DT primers for template conversion and cDNA generation (17). The analysis of sequencing data is the last part of SCS. Uneven coverage in the data and chimeric reads with great regularity are surprisingly common (18). Standardizing the nucleic acid library and performing hybrid analysis for sequencing data with high genetic similarity using SmashCell, Velvet-SC, and SPAdes software are strategies to provide more reliable results (19).

## Single-Cell Sequencing in Cancer: Lessons Learned

SCS techniques have emerged as promising approaches to dissect human tumors at the resolution of individual cells and therefore represent useful tools for deciphering cancer biology. Here, we summarize current SCS data from various human cancers.

### Research Progress of Single-Cell Sequencing in Lung Cancer

Lung cancer is a very heterogeneous disease composed of multiple unique histologic subtypes that harbor distinct molecular signatures (20). ITH in preneoplastic lesions is often addressed in the context of missed diagnosis, limited therapeutic success, high mortality, and poor prognosis of lung cancer (21, 22). Tracing a gene map of precursor lesions in lung nodules was crucial for gaining a better intelligence about the mechanisms underlying the occurrence and development of early lung cancer. A single-cell transcriptome atlas of lung adenocarcinoma featuring ground-glass nodule adenocarcinoma (GGN-ADC) was created using scRNA-seq, and in the atlas, eight cell types (cancer cells, endothelial cells, fibroblasts, T cells, B cells, natural killer cells, mast cells, and myeloid cells) were found in the TME (23). The cell cycle and the nuclear factor (NF)-κB, Toll-like receptor (TLR), and vascular endothelial growth factor (VEGF) signaling pathways, which are related to cell proliferation, were found to be downregulated in GGN-ADC compared with solid adenocarcinoma (SADC) cancer cells (24). Similarly, activation of the phosphoinositide 3-kinase/protein kinase B (PI3K/AKT), hypoxia-inducible factor-1 (HIF-1), and VEGF signalling pathways, which accelerates angiogenesis, was attenuated in GGN-ADC endothelial cells (25). However, immunosuppressive pathways were activated in T cells derived from GGN-ADC, and the cytotoxicity of NK cells was more robust. In addition, macrophages tended to become M1 polarized, and mast cells were more enriched in GGN-ADC (26). These findings suggest that imbalanced activation of innate and adaptive immune responses not only makes immune escape possible but also easily induces an inflammatory storm. Transcriptome data from GGN-ADC-derived cells were also updated with data from scRNA-seq using module G64. These cells can be clearly divided into two groups according to the upregulated or downregulated expression of cell cycle-related genes, which is considered a guideline to identify surgical patients with lung adenocarcinoma in The Cancer Genome Atlas (TCGA) database (27). In summary, it has been well documented using scRNA-seq that cancer cells and the TME together preferentially contribute to the growth of GGN-ADC over SADC. Encouragingly, the dissimilarities within the TME between GGN-ADC and SADC provide clear ideas for elucidating why GGN-ADC remains stable and has better survival.

ScRNA-seq has also revealed heterogeneous tumor and immune cell populations in the TME of early-stage lung adenocarcinomas harboring EGFR mutations, among which myeloid cells, T cells, tumor-associated macrophages (TAMs), and dendritic cells (DCs) are prominent. In particular, DCs in lung adenocarcinomas are mainly CD1C^+^, implying their function in the inhibition of effector T cells and the promotion of regulatory T cells (Tregs). These tumor-infiltrating T cells demonstrate exhausted and Treg features. Furthermore, TAMs display protumoral functions without M1 or M2 polarization (28). By assessing tumor-infiltrating myeloid cells (TIMs) in patients with non-small-cell lung cancer (NSCLC) using scRNA-seq, 25 TIM-specific genes, such as TREM2, CD81, MARCO, and APOE, were consistently identified, and their expression in bone marrow cells from different species was extremely heterogeneous (29, 30). Dimensionality cluster analysis of scRNA-seq data from NSCLC patients identified undetectable expression of TNFRSF9, which suggested that the T cells were suffering from exhaustion. A low abundance of TNFRSF9 in these “pre-exhausted” cells was positively associated with a worse prognosis of NSCLC (31). These findings highlight the role of differences among tumor-infiltrating lymphocytes (TILs), the dysfunction of which happens to be a biomarker of immunotherapy response in lung cancer.

Tumor invasion and metastasis are responsible for the majority of deaths in lung cancer (32). The subtypes of lung cancer cells deviating from the differentiation track were identified using scRNA-seq, and the dynamic changes in stromal and immune cells suggested tumor metastasis (33). Circulating tumor cells (CTCs) may be an initiator of metastasis and induce poor prognosis in lung cancer (34). Characteristic exon-related indel polymorphisms in CTCs have also been identified through scRNA-seq. The copy number variation (CNV) in each CTC is highly consistent regardless of the cancer subtype, thus indicating that CNV is a dominant factor influencing tumor metastasis (35). Overall, the characterization of CTCs has provided fascinating insights into the heterogeneity of lung cancer, which may raise new ideas for improving the diagnosis and treatment of lung cancer.

Chemotherapy has been approved as a standard treatment for lung cancer; unfortunately, tumor metastasis and local recurrence are becoming increasingly common due to chemoresistance (36). Emerging scRNA-seq data from biopsy specimens from lung cancer patients treated with or without chemotherapy indicate that the surviving tumor cells in the TME exist in an intricate and dynamic ecosystem and are typically activated in the alveolar pathway (37). Another study showed that patient-derived xenograft (PDX) cells with notable expression of KRAS^G12D^ were resistant to chemotherapy, thus confirming that KRAS mutation status and a risk score can be used to predict the outcome in lung cancer (38). Therefore, studies discerning the cell distribution in lung cancer *via* SCS have suggested that drug-resistant subpopulations are more resistant to the action of chemotherapeutic drugs, even influencing the TME to benefit themselves.

### Application of Single-Cell Sequencing in Hepatocellular Carcinoma

As the third leading cause of cancer-related deaths worldwide, hepatocellular carcinoma (HCC) is difficult to combat due to its high degree of malignancy and poor prognosis (39). Immunotherapy has brought hope for the treatment of HCC (40). To assess the immune microenvironment in HCC and identify innovative biomarkers for immunotherapy of HCC, T cells in HCC tissues were analyzed by scRNA-seq, and T-cell subpopulations were reviewed in a transcriptional map. A large number of CD8^+^ T cells and Tregs were found. Layilin inhibited the activation of CD8^+^ T cells but activated Tregs and thus might be a potential agent for immunotherapy in HCC (41). These findings demonstrated that CD8^+^ T-cell failure can be a hallmark of hepatocarcinogenesis, which may be instrumental for the development of novel approaches for effective immunotherapy for HCC.

Non-alcoholic steatohepatitis (NASH) represents one of the leading causes of HCC, but the underlying mechanisms associated with the carcinogenesis of NASH-related HCC have not been clarified (42). ATAC-seq identified chromatin-accessible regions in NASH-derived HCC tissue samples, and 139 upregulated and 60 downregulated genes were specifically found. Interestingly, 15 of the 139 upregulated genes had accessible chromatin sites within 5 kb of the transcription start site (TSS), including APOA4, SERPINE1, IGFBP1, ANXA2, and TUBB2a. The upregulation of these genes was found to be associated with the enrichment of transcription factors (TFs), especially NFATC2, and histone H3K4me1 and H3K27ac gene transcription-activating marks in chromatin-accessible regions (43). These data highlight the role of chromatin accessibility perturbations in reshaping the chromatin landscape in NASH-related HCC. Hepatitis B virus (HBV) represents one of the most well-known etiologic factors of HCC (44). The mechanism by which HBV regulates both host and viral gene transcription involves chromatin modification mediated by an RNA helicase, DEAD-box protein 5 (DDX5). ATAC-seq was performed in which the chromatin accessibility of wild-type *DDX5* cells *vs. DDX5^KD^* cells was compared. ATAC-seq of DDX5^KD^ and wild-type DDX5 cells further identified that DDX5 inhibits Wnt signaling by affecting chromatin accessibility of genes involved in Wnt signaling, and DDX5 may be a negative regulator of Wnt signalling and hepatocyte reprogramming in HCC (45).

CD24, CD133, and epithelial cell adhesion molecule (EpCAM) have been identified as markers of hepatocarcinoma stem cells (HSCs), which have been determined to have ITH (46). ScRNA-seq data have verified that CD24^+^/CD44^+^ cells are enriched in EpCAM^+^ HSC populations (47). Therefore, the differentially expressed genes between HSC subtypes affect the prognosis of HCC. In addition, HSC-induced transcriptome diversity is tightly related to ITH generation, thus providing new ideas for biomarkers for predicting prognosis and treatment response in HCC.

ScRNA-seq data from CTCs of HCC patients have attracted much attention. Overexpression of IGF2, a gene overexpressed in 20% of early HCCs and recently characterized as an HCC epidriver, was detected in CTCs (48, 49). Thus, scRNA-seq of CTCs introduced the application of liquid biopsy in the early screening and diagnosis of HCC based on the non-mutated druggable genomic aberrations such as IGF2 overexpression. CTC count ≥16 and mesenchymal CTC (M-CTC) percentage ≥2% prior to resection have been identified as significantly associated with early recurrence, multi-intrahepatic recurrence, and lung metastasis. Gene expression profiling has identified a scene in which a concomitant increase in mesenchymal marker expression (vimentin and Twist) and reduced epithelial marker expression (EpCAM and E-cadherin) are exclusively found in CTCs (50). Moreover, CTCs have been isolated from the peripheral vein, hepatic vein, inferior vena cava, and portal vein of HCC patients with early metastasis. Gene expression profiling of these CTCs suggests that they are undifferentiated compared with epithelial−mesenchymal transition (EMT)-like cells, thus indicating that CTCs were changed by the TME and participated in ITH induction in metastatic HCC (51). Collectively, systematic adhesion of CTCs shapes EMT-driven HCC metastasis, hence suggesting the utility of individualized treatments for HCC.

### Single-Cell Sequencing: A Contemporary Approach in Colorectal Cancer

ScRNA-seq analysis of single colorectal cancer and adjacent normal intestinal gland cells has been carried out. The results confirmed that colorectal cancer cells possessed a wide range of genetic differences, and the levels of DNA methylation in tumor cells also varied (52). Another important finding from CROP-seq is that colorectal adenoma and colorectal cancer are of the same origin based on ITH and can be divided into different subclonal types based on the presence of non-random somatic mutations in the G protein-coupled receptor (GPCR), intracellular PI3K/AKT, and fibroblast growth factor receptor (FGFR) signaling pathways (53). Primary and metastatic tumor cells and CTCs in colorectal cancer have also been assessed using scRNA-seq. APC-, KRAS-, and PIK3CA driver mutations in the corresponding CTCs were revealed and were present in the premetastatic lesions (54). Alternatively, two vastly different cancer-associated fibroblast (CAF) profiles were identified in the TME of primary colorectal cancer, and mesenchymal markers such as vimentin and N-cadherin were upregulated only in the “CTC-CAF” subgroup, suggesting the utility of EMT marker-directed diagnostic methods and targeted therapies (55). Thus, the roles of ITH in tumor evolution and metastasis of colorectal cancer have been well validated.

### Application of Single-Cell Sequencing in Esophageal Cancer and Gastric Cancer

SCS data in esophageal cancer are scarce, and studies have mainly focused on chemoresistance. The paclitaxel-resistant esophageal cell line KYSE-30 was successfully induced through exposure to low-dose paclitaxel. Results acquired from scRNA-seq advocated for the presence of differential expression of KRT19 between KYSE-30 and parental esophageal cancer cells, thus suggesting that there is inherent paclitaxel resistance in esophageal cancer (56). KYSE-30 cells are also characterized by noteworthy expression of genes related to the ubiquitin proteasome pathway, and downregulation of the expression of such genes provokes the HIF-1 signaling pathway, ultimately resulting in paclitaxel resistance in esophageal cancer (56). However, additional SCS data from esophageal cancer are needed.

An unbiased transcriptome-wide scRNA-seq analysis of gastric cancer (GC) cells from gastric adenocarcinoma patients identified five subgroups with distinct gene expression profiles, marking the beginning of a new era in delineating the cellular heterogeneity in GC. Among the subgroups, three subgroups exhibited different differentiation grades, which corresponded well to the histopathological features of Lauren’s subtypes. Interestingly, the other two subgroups displayed unique transcriptome features. One subgroup expressing chief-cell markers (e.g., LIPF and PGC) and RNF43 and with Wnt/β-catenin signaling pathway activation was consistent with the previously described entity fundic gland-type GA (chief cell-predominant, GA-FG-CCP). The other subgroup specifically expressed immune-related signature genes (e.g., LY6K and MHC II) and had Epstein−Barr virus infection. In addition, non-malignant epithelium analysis using scRNA-seq has offered molecular evidence for the potential transition of gastric chief cells into MUC6^+^/TFF2^+^ spasmolytic polypeptide-expressing metaplastic cells (57). Hereditary diffuse gastric cancer (HDGC) is a cancer syndrome caused by germline variants in CDH1, the gene encoding the EMT marker E-cadherin, but the mechanisms through which CDH1 loss initiates HDGC have not been determined. In an automated scRNA-seq analysis, gastric epithelium with CDH1 deletion was found to be enriched for stromal cells, macrophages, dendritic cells (DCs) and Tregs. These TME-exclusive cells with distinct expression of extracellular matrix components and cytokeratin 7 (CK7) decreased transcriptional heterogeneity. In particular, the macrophages did not conform to the binary M1/M2 paradigm, and gastric DCs had a gene expression program unique from that of peripheral blood mononuclear cell (PBMC)-derived DCs. Moreover, TME-specific helper T cells, cytotoxic T cells, Tregs, and NK cells were found to express multiple immune checkpoint and costimulatory molecules (58). In addition, CDH1 loss resulted in shifts along the squamous differentiation trajectory, as well as the aberrant expression of the KRT7 gene (encoding the CK7 protein), which was identified as the top candidate leading to gastrointestinal epithelial invasion and early metastasis in CDH1 carriers (59). Chromatin accessibility has been identified as one of the most relevant genomic characteristics associated with oncologic functions at a specific site, providing the structure for transcription TFs binding to regulate multiple genes, thus driving cancer progression and invasion (60). Dysregulated chromatin accessibility is critical for GC metastasis, but the mechanisms remain unclear. ATAC-seq suggested that bromodomain-containing protein 4 (BRD4) is a crucial regulator of chromatin remodeling that promotes GC progression by regulating multiple genes. JQ1, also known as a small-molecule inhibitor of BRD4, drives chromatin accessibility changes in GC cells, and differentially accessible regions are highly enriched for RUNX2-binding motif. JQ1 suppressed GC metastasis by downregulating chromatin accessibility, and JQ1-inducing inhibition of GC cell metastasis is dependent on EMT suppression caused by alleviated activation of the RUNX2/NID1 pathway (61). Therefore, GC cells trigger TME-exclusive intercellular communication (leading to EMT), thus affecting distant metastasis.

### Unraveling the Biological Mechanisms and Shifting Paradigms in Pancreatic Cancer With Single-Cell Sequencing

Pancreatic cancer is a lethal disease with extremely poor prognosis and is characterized by high heterogeneity of tumor cells and the TME, which lead existing therapies to offer only limited effectiveness (62). Cystomas are believed to be involved in inciting the development of pancreatic cancer. Of cystomas, intraductal papillary Mucinous neoplasms (IPMNs) are the most commonly identified, but there are no reliable markers to distinguish inert from invasive IPMNs (63). ScRNA-seq was used to trace precancerous lesions with 5,403 cells from low-grade IPMN (LGD), high-level IPMN (HGD), and pancreatic cancer specimens. Heterogeneity in epithelial cells of LGD specimens was found; this caused overexpression of the oncogene transcriptome in LGD specimens, and tumor growth-suppressing pathways were evidently activated at the same time. However, these tumor growth-suppressing pathways were repressed in HGD specimens and were inactivated in pancreatic cancer specimens (64). Acinar-to-ductal metaplasia is an early event in the initiation of pancreatic cancer but is generally poorly understood. SnRNA-seq indicates that the expression of the PDAC-associated oncogene GNASR201C more effectively produces cystic growth in ductal organoids than in acinar organoids, whereas KRAS^G12D^ more effectively models cancer *in vivo* when expressed in acinar organoids than when expressed in ductal organoids. KRAS^G12D^, but not GNASR201C, induces acinar-to-ductal metaplasia-like changes in culture and *in vivo* (65). Furthermore, the transcription factor ONECUT2 was identified as a putative driver of early progression of pancreatic cancer whose role in the activation of the KRAS pathway was sufficient to induce acinar hyperplasia, although the neoplastic lesions developed focally (66). Regardless, SCS has identified a renewable source of ductal and acinar organoids for modeling exocrine development and diseases, and lineage tropism and plasticity in terms of oncogene action in the human pancreas have been well understood using SCS.

Most patients with pancreatic cancer will relapse within 4 years despite early diagnosis and surgical resection, suggesting that early micrometastasis develops silently (67). The response to adjuvant therapies is heterogeneous, with metastasis typically resulting due to mutations in the CDKN2A, SMAD4, TP53, and KRAS genes (68). A TSCS study described the spatial characteristics of metastasis of pancreatic cancer, in which a cutoff of at least 10 CTCs was established as a lower limit for reliable KRAS mutational analysis of CTCs (69). More recently, a variety of cell subsets in primary and metastatic tumor tissues were identified through scRNA-seq, among which CTCs with an EMT phenotype were located in only metastatic lesions and predicted a worse prognosis of pancreatic cancer (70). Another meaningful study using scRNA-seq identified heterogeneity between primary and metastatic lesions, confirming that BIRC5 expression was massively upregulated in metastatic CTCs. YM155 administration in a model with BIRC5 deletion shrank the metastatic foci in SW1990-bearing mice, thus indicating that BIRC5 in CTCs is a key coordinator of the metastasis of pancreatic cancer (71).

A gene map of fibroblasts in pancreatic cancer was drawn using scRNA-seq analysis, in which transforming growth factor (TGF)-β1 and LRRC15 expression were prominent. These fibroblasts appeared insensitive to anti-programmed death ligand 1 (PD-L1) therapy (72). Previous studies have shown that CD47 expression tempers the phagocytosis of macrophages (73), but little is known about CD47 expression in fibroblasts and its implication in pancreatic cancer. In fact, it has been found using scRNA-seq that anti-CD47 treatment leads to the chemotaxis of pro-inflammatory fibroblasts and the diminishment of immunosuppressive macrophages (74). More strikingly, the inflammatory fibroblasts overexpressed CXCL12, and these CXCL12-expressing fibroblasts were found to encourage the immune escape of pancreatic cancer cells (75). Therefore, these “unconventional” fibroblasts were reprogrammed to induce an immunotolerance framework in pancreatic cancer.

### Understanding Breast Cancer With Single-Cell Sequencing

Breast cancer originates from ducts and epithelial cells and gradually develops from hyperplasia to atypical hyperplasia, *in situ* (adeno)carcinoma, and finally early and advanced invasive carcinoma (76). As early as 2011, the evolution of multicellularity in breast cancer was deduced, and three subpopulations in different clones were identified through scRNA-seq. An unexpectedly rich subgroup, namely, “pseudodiploid” cells, was reported. These “pseudodiploid” cells do not migrate to the site of disease progression and do not follow the path of tumor progression, which illustrates that breast cancer cells grow continuously with clonal expansion (8). The continuous growth of clonal subpopulations guides cell differentiation into five subtypes in accordance with the expression of estrogen receptor (ER), progesterone receptor (PR), and human epidermal growth factor receptor 2 (HER2) (77). Determining the changes in gene copy number with SCS is helpful for distinguishing cancer cells from non-cancer cells. Non-cancer cells include T cells, B cells, and macrophages. Both T cells and macrophages are regarded widely as immunosuppressive: Tregs can experience immune anergy and exhibit co-mutation of CTLA4, TIGHT, and GITR genes, and macrophages can take on an M2 phenotype (78, 79). Furthermore, aberrant expression of CDH1 and PVRL2, NK cell inhibitory receptor ligands, plays a very important role in the immune evasion of breast cancer cells. ATAC-seq data have demonstrated that CDH1 is positively regulated by TFs such as NFATC2, XBP1, YY1AR, GTF2I, IRF2, and NF1, and variations in CDH1 expression are mainly caused by CNV. However, CNV and the mRNA level of PVRL2 are very weakly correlated, and high expression of PVRL2 may also be induced by increases in TFs including FOXP3, GTF2I, YY1, NR3C1, SP1, TFAP2A, AR, ESR1, PAX5, and TP53 (80). This evidence indicates that ITH in breast cancer is boosted by the combined action of tumor cells and immune cells in the TME.

Somatic mutations provide a basis for the selection of clones with an advantage and imply that there is a causal relationship of oncogene dynamics with breast cancer (81). Somatic TP53 and PIK3CA driver mutations have been found to originate early in the progression of breast cancer (82). Changes in somatic copy number and the mutated genes encoding exogenous proteins in breast cancer have been identified, and strong correlations between mutations and histological grade have been corroborated using scRNA-seq (83, 84). In conclusion, SCS has contributed to a novel understanding of cell-level somatic mutation information that can facilitate investigation of cell heterogeneity.

Ductal carcinoma *in situ* (DCIS) constitutes the most common form of early breast cancer, but the metastasis of DCIS remains poorly understood (85). TSCS has been used to assess changes in gene copy number and to track the spatial information and clonal evolution of single tumor cells sorted from metastatic DCIS-IDC patients. A common genomic lineage was identified between *in situ* and invasive subpopulations, and most mutations and copy number aberrations evolved in the premetastatic lesion before tumor metastasis (86). The clones cooperated with each other to escape from the basement membrane and migrated to adjacent tissues to establish invasive carcinoma (87). Moreover, the heterogeneity between the primary tumor and metastases in lymph nodes was assessed using scRNA-seq, and a novel cell subpopulation called CXCL14 cancer cells was identified in the positive lymph nodes of breast cancer patients. ATAC-seq of the positive and negative lymph node samples further revealed the chromatin accessibility profile and identified potential TFs related to CXCL14 cancer cells, including ZNF467, bZIP, EBF1, and PIT1, in the lymph node metastases of breast cancer (88).

Triple-negative breast cancer (TNBC) is the most invasive and malignant type of breast cancer, and TNBC lacks ER, PR, and HER2 expression (89). Chemotherapy remains the standard therapeutic approach for TNBC, with neoadjuvant chemotherapy (NAC) being preferred (90). However, nearly half of the patients with TNBC have NAC resistance, leading to overall refractoriness of the disease. Two distinct clonal dynamic patterns were identified in NBC patients with NAC using scRNA-seq: “regressive” and “persistent.” NAC administration eliminated almost all the tumor cells in “regressive” patients, allowing only autosomal diploid cells such as fibroblasts and immune cells to thrive. In contrast, there were a large number of residual tumor cells in “persistent” patients whose genotypes were reprogrammed by NAC (91). Furthermore, the gene profile was selectively modulated, and the transcription profile in TNBC patients treated with docetaxel and epirubicin was modulated. However, a series of chemoresistance-driving genes had shown increased expression before NAC administration, implying that chemoresistance was preexisting (91). After employing scChIP-seq for breast cancer PDX samples, tumor heterogeneity at the chromatin level was characterized. Notably, loss of repressive chromatin marks such as H3K27me3 is associated with stable transcriptional repression of indicated genes that probably cause resistance to NAC (92). ScRNA-seq data have laid a foundation for clarifying the role of tissue-resident memory (TRM) cells differentiated from CD8^+^ T cells in breast cancer. These CD8^+^ TRM cells show remarkable expression of fatty acid-binding protein 4 (FABP4) and FABP5, which have received public attention for their role as immune checkpoint proteins that likely influence survival in TNBC patients (93). Overall, the development of SCS, by which genomic, transcriptomic, and epigenetic information from TNBC cells can be extracted, has been timely and has enabled the effective identification of unique mutations influencing ITH in rare subpopulations and an understanding of the evolutionary lineage.

### Beginning of a New Era: Mapping Niches in Hematological Malignancies Using Single-Cell Sequencing

The last decade has witnessed great advances in our understanding of the genetic basis of childhood T-cell acute lymphoblastic leukemia (T-ALL), but the effects of gene mutations in progenitor cells of T-ALL are ambiguous (94). In this regard, T-ALL mutations have been clarified using scRNA-seq. Specifically, loss of the chromosome 9p21 fragment was found to be an intermediate event, while *Notch1 mutation* was usually identified as a late event. Furthermore, STIL-TAL1 gene fusion and CDKN2A gene loss were identified as early and major events (95, 96).

Diffuse large B-cell lymphoma (DLBCL) is one of the most common subtypes of non-Hodgkin’s lymphoma (NHL). ScRNA-seq was used to reveal the phenotypic heterogeneity in DLBCL, revealing the close association of MHC II heterogeneity with DLBCL initiation (97). Vitreoretinal lymphoma (VRL) is a rare ocular malignant tumor (98). ScRNA-seq confirmed that the *MyD88 L265P mutation* accounts for more than 60% of all VRL mutations and can be used as a useful marker for the diagnosis of VRL (99–101). Mantle cell lymphoma (MCL) is an incurable form of NHL (102). MCL subsets with obvious TME heterogeneity have been identified through the implementation of scRNA-seq. Immune escape and drug resistance mechanisms in MCL are related to drug metabolism, DNA damage repair, apoptosis, and survival promotion (103).

Cutaneous T-cell lymphoma (CTCL) is a heterogeneous group of lymphoproliferative disorders, including Sézary syndrome, which has a poor prognosis and unclear pathogenesis (104). ScRNA-seq was performed and revealed that Foxp3^+^ malignant T cells induce the metastasis of GATA3^+^ and IKZF2^+^ clonal tumors, and Foxp3 might be a candidate for predicting early CTCL (105).

Langerhans cell histiocytosis (LCH), a spectrum of diseases formerly known as histiocytosis X, is characterized by diverse clinical manifestations involving bone, skin, lung, and pituitary tissue (106). Detailed maps of LCH lesions have been generated with scRNA-seq data, which confirmed the evolution of LCH and provided ground-breaking indicators for personalized treatment of LCH (107).

In conclusion, the potential roles of SCS in the diagnosis and treatment of hematological malignancies have been summarized, but more efforts should be made to highlight the advantages of SCS from a clinical standpoint.

## Conclusion and Prospects

**Figure 1:**
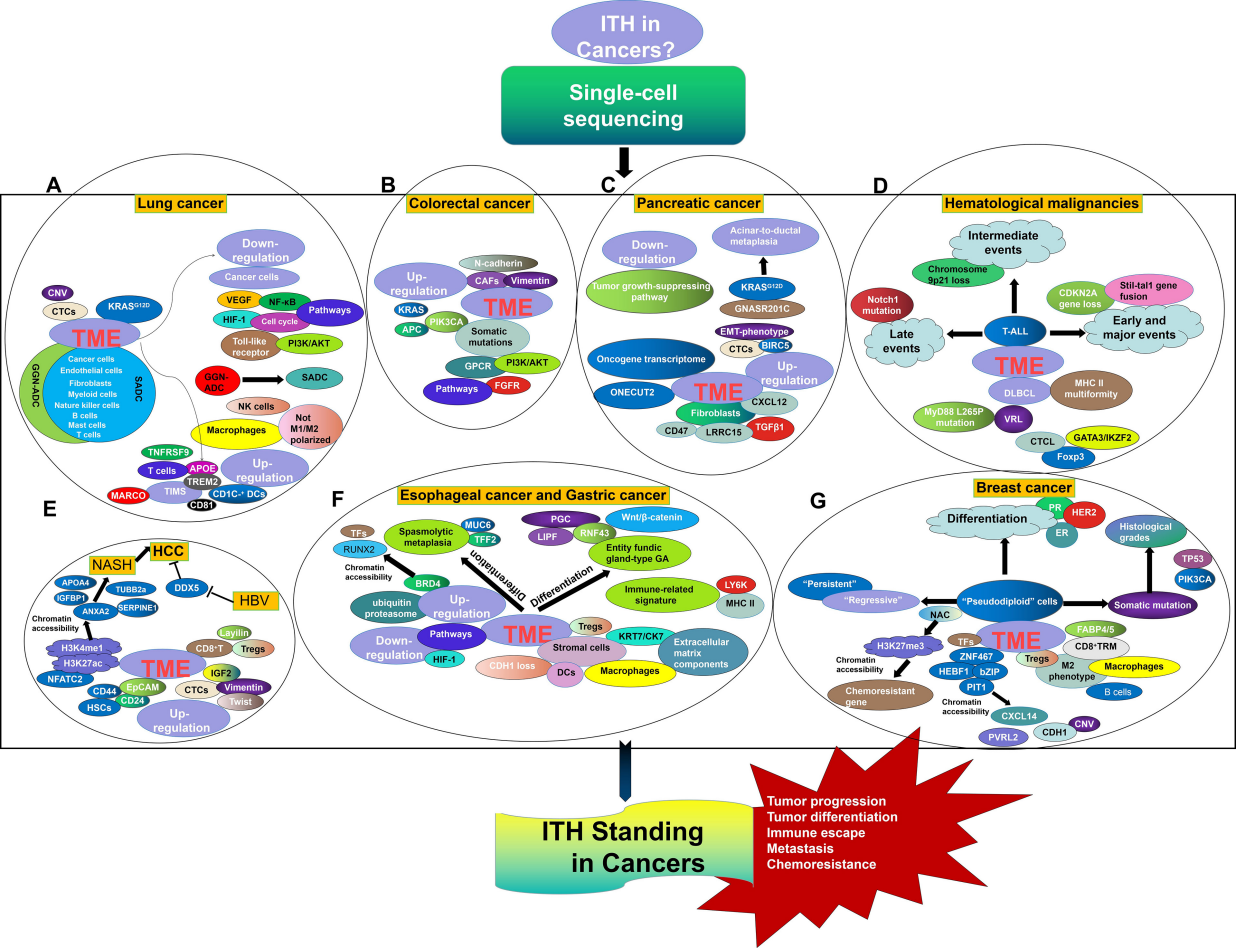
ITH Standing in Lung cancer **(A)**, Colorectal cancer **(B)**, Pancreatic cancer **(C)**, Hematological malignancies **(D)**, HCC **(E)**, Esophageal cancer and gastric cancer **(F)**, and Breast cancer **(G)** from the perspective of SCS.

SCS has obviously laid a foundation for a renewed understanding of tumor initiation and evolution. Here, the precise characterization of single-cell programs in lung cancer, HCC, colorectal cancer, esophageal cancer, GC, pancreatic cancer, breast cancer, and hematological malignancies was reviewed, highlighting the importance of cellular biology in cancer biology (**Figure 1**). However, there are many future challenges for this field that should not be ignored. In particular, the accuracy of targeted single-cell isolation, purification, and measurement is critical for ensuring the validity of SCS data. It is truly difficult to isolate the target cells and prevent them from being cross-contaminated with other cells. SCS technological developments are opening multiple new avenues, and cryopreservation has been shown to be suitable for cell capture and library preparation (108). Moreover, an automated single-cell analysis and isolation system was developed that showed superior noninvasive isolation of a single cell with the most favorable properties from arrays containing 10^5^ cells comparing with a fluorescence-activated cell sorter. Such automated technology can be used for high-throughput screening for single-cell isolation with targeted labeling (109). In addition, amplification failure and allelic contamination with nonspecific products are frequent in SCS. Improvements in strategies such as template switching, second-strand synthesis for *in vitro* transcription mediated by RNaseH/DNA polymerase I, and poly(A) tagging have led to better quantitative performance of SCS (110). Additionally, SCS prevalence has suffered due to the substantial cost of bioinformatics analysis, and it is more difficult for patients who have already borne the heavy burden of cancer to agree to undergo SCS analysis. Fortunately, the development of alternate scRNA-seq platforms, in which thousands of cells are profiled, is enabling the exploration of tumor subpopulations, including those with adaptations underlying chemoresistance and metastatic advancement (111). Moreover, the Human Cell Atlas (HCA) project is attempting to conduct phenotyping at the single-cell level, hence generating cellular maps of cell lineages, organs, and organisms (112). Thus, deciphering the genome of a single cell will eventually be applicable in ordinary cancer patients, as many efforts have been made to continuously optimize single-cell separation and single-cell genome amplification technologies. The place of SCS in understanding ITH development and effects as they apply to tumor development, metastasis, chemoresistance, and immune escape cannot be discounted.

## Author Contributions

JL contributed study concept design to this study. NY, XL, and MC consulted indicated references and refined interpreted information from acquired data. QG wrote the first draft of the article. All authors contributed to the article and approved the submitted version.

## Funding

This work was supported by the Shandong Provincial Natural Science Foundation (ZR2020QH362).

## Conflict of Interest

The authors declare that the research was conducted in the absence of any commercial or financial relationships that could be construed as a potential conflict of interest.

## Publisher’s Note

All claims expressed in this article are solely those of the authors and do not necessarily represent those of their affiliated organizations, or those of the publisher, the editors and the reviewers. Any product that may be evaluated in this article, or claim that may be made by its manufacturer, is not guaranteed or endorsed by the publisher.

## Glossary

**Table:**

SCS	single-cell sequencing
ITH	intratumor heterogeneity
TME	tumor microenvironment
HGP	Human Genome Project
NGS	next-generation sequencing
TGS	third-generation sequencing
ScRNA-Seq	single-cell RNA sequencing
WGS	whole-genome sequencing
WTS	whole-transcriptome sequencing
ATAC-Seq	assay for transposase-accessible chromatin with sequencing
SnRNA-Seq	single-nucleus RNA sequencing
ScChIP-Seq	single-cell chromatin immunocleavage sequencing
TSCS	topographic single-cell sequencing
CROP-Seq	CRISPR droplet sequencing
LCM	laser-capture microdissection
FACS	fluorescence-activated cell sorting
IMS	immunomagnetic separation
ROT	Raman optical tweezers
TAS	micro-total analysis system
WGA	whole-genome amplification
WTA	whole-transcriptome amplification
PCR	polymerase chain reaction
DOP-PCR	degenerative-oligonucleotide-primed PCR
MDA	multiple displacement amplification
MLBAC	multiple annealing- and looping-based amplification cycle
IVT	*in vitro* transcription
GGN-ADC	ground-glass nodule adenocarcinoma
TLR	Toll-like receptor
VEGF	vascular endothelial growth factor
SADC	solid adenocarcinoma
HIF-1	hypoxia-inducible factor-1
TIMs	tumor-infiltrating myeloid cells
TAMs	tumor-associated macrophages
NSCLC	non-small-cell lung cancer
TILs	tumor-infiltrating lymphocytes
CTCs	circulating tumor cells
CNV	copy number variation
PDX	patient-derived xenograft
HCC	hepatocellular carcinoma
Tregs	regulatory T cells
NASH	non-alcoholic steatohepatitis
TSS	transcription start site
HBV	hepatitis B virus
DDX5	DEAD-box protein 5
HSCs	hepatocarcinoma stem cells
EMT	epithelial–mesenchymal transition
GPCR	G protein-coupled receptor
FGFR	fibroblast growth factor receptor
CAFs	cancer-associated fibroblasts
GC	gastric cancer
HDGC	hereditary diffuse gastric cancer
BRD4	bromodomain-containing protein 4
IPMNs	intraductal papillary myxomas
PD-L1	programmed death ligand 1
ER	estrogen receptor
PR	progesterone receptor
TFs	transcription factors
HER2	human epidermal growth factor receptor 2
DCIS	ductal carcinoma *in situ*
TNBC	triple-negative breast cancer
NAC	neoadjuvant chemotherapy
FABP4	fatty acid-binding protein 4
TRM	tissue-resident memory
T-ALL	T-cell acute lymphoblastic leukemia
DLBCL	diffuse large B-cell lymphoma
NHL	non-Hodgkin’s lymphoma
VRL	vitreoretinal lymphoma
MCL	mantle cell lymphoma
CTCL	cutaneous T-cell lymphoma
LCH	Langerhans cell histiocytosis
HCA	Human Cell Atlas
